# Atrial myxoma-related embolic stroke: two distinct embolic mechanisms and clinical implications in young adults (a case series)

**DOI:** 10.1186/s12883-026-05165-0

**Published:** 2026-07-10

**Authors:** Cheng-zhi Xing, Si-ping Li, Chang-yong Dao, Xian-wen Wei

**Affiliations:** 1 Department of Neurology, Pu’er People’s Hospital, Pu’er, Yunnan Province China; 2 Department of Ultrasound Medicine, Pu’er People’s Hospital, Pu’er, Yunnan Province China

**Keywords:** Atrial myxoma, Mechanical thrombectomy, Tumor embolism, Multisystem embolism, Dual embolism mechanism, Young stroke

## Abstract

**Background:**

Cardiac myxoma can lead to stroke via two distinct embolic mechanisms: embolism from tumor fragments or thromboembolism from the tumor surface. Pathological confirmation of the embolus is rarely conducted; however, the pathological features of stroke have implications for treatment.

**Case series report:**

We present two young Chinese patients with acute ischemic stroke subsequent to atrial myxoma, whose stroke was caused via distinct embolic mechanisms. The first patient exhibited isolated basilar artery occlusion due to secondary thrombosis. This patient underwent successful computed tomography(CT)-guided mechanical thrombectomy 11 h after symptom onset. The second patient exhibited the combination of cerebral and peripheral embolism due to direct shedding of tumor emboli and was subjected to sequential endovascular treatment. Both patients achieved complete recanalization and favorable 3-month functional outcomes after tumor resection. These cases highlight phenotypic heterogeneity, differences in embolic mechanisms, and the value of integrated imaging, pathological examination, cardiac assessment, and individualized therapy in atrial myxoma-related stroke.

**Conclusion:**

Atrial myxoma-associated strokes involve two distinct mechanisms of embolism that may affect secondary prevention and follow-up strategies. Accurate differentiation relies on the pathological examination of embolic material, which requires coordinated multidisciplinary care to develop personalized treatment.

## Introduction

Ischemic stroke affects 20%-45% of patients with cardiac myxoma [1]. However, the underlying mechanisms have not been fully explored, and the associated risk factors for cardiac myxoma-related stroke remain poorly defined. Various processes may contribute to the development of stroke in patients with cardiac myxoma. A recent review article suggested two different mechanisms: embolism originating from the tumor, and metastatic spread [2]. However, the first mechanism may result in heterogeneous pathological features of thrombus. This article aimed to highlight the importance of thrombus pathological examination and elucidate the underlying mechanisms, which are essential in selecting the optimal management strategy for cardiac myxoma-related stroke.

## Case presentation

### Patient 1 case description

A 44-year-old man with a 20-pack-year smoking history developed acute dizziness, nausea, vomiting, dysarthria, left-sided numbness, and weakness. Initial examination revealed horizontal nystagmus, left tongue deviation, and limb ataxia. The National Institutes of Health Stroke Scale (NIHSS) score was 7 (I2 = 1, I4 = 1, I5a = 1, I6a = 1, I7 = 1, I8 = 1, and I10 = 1).

Neuroimaging at 8.5 h after symptom onset demonstrated basilar artery terminus occlusion on a computed tomography angiogram (CTA) (Fig. 1A). Quantitative cerebral perfusion analysis was conducted using an automated RApid processing of PerfusIon and Diffusion (RAPID) post-processing algorithm for assessing the pathophysiology of acute ischemic lesions.Irreversible infarct core was delineated as voxels with relative cerebral blood flow (rCBF) less than 30% of homologous contralateral brain parenchyma, yielding a core volume of 4.22 mL. Tissue at risk of infarction was defined based on a Tmax threshold of > 6.0 s, which indicated a total volume of 106.06 mL.Perfusion mismatch volume, calculated as the difference between Tmax > 6.0 s volume and infarct core volume, was 101.84 mL, corresponding to a mismatch ratio of 25.13 (Fig. 1B).

**Fig. 1:**
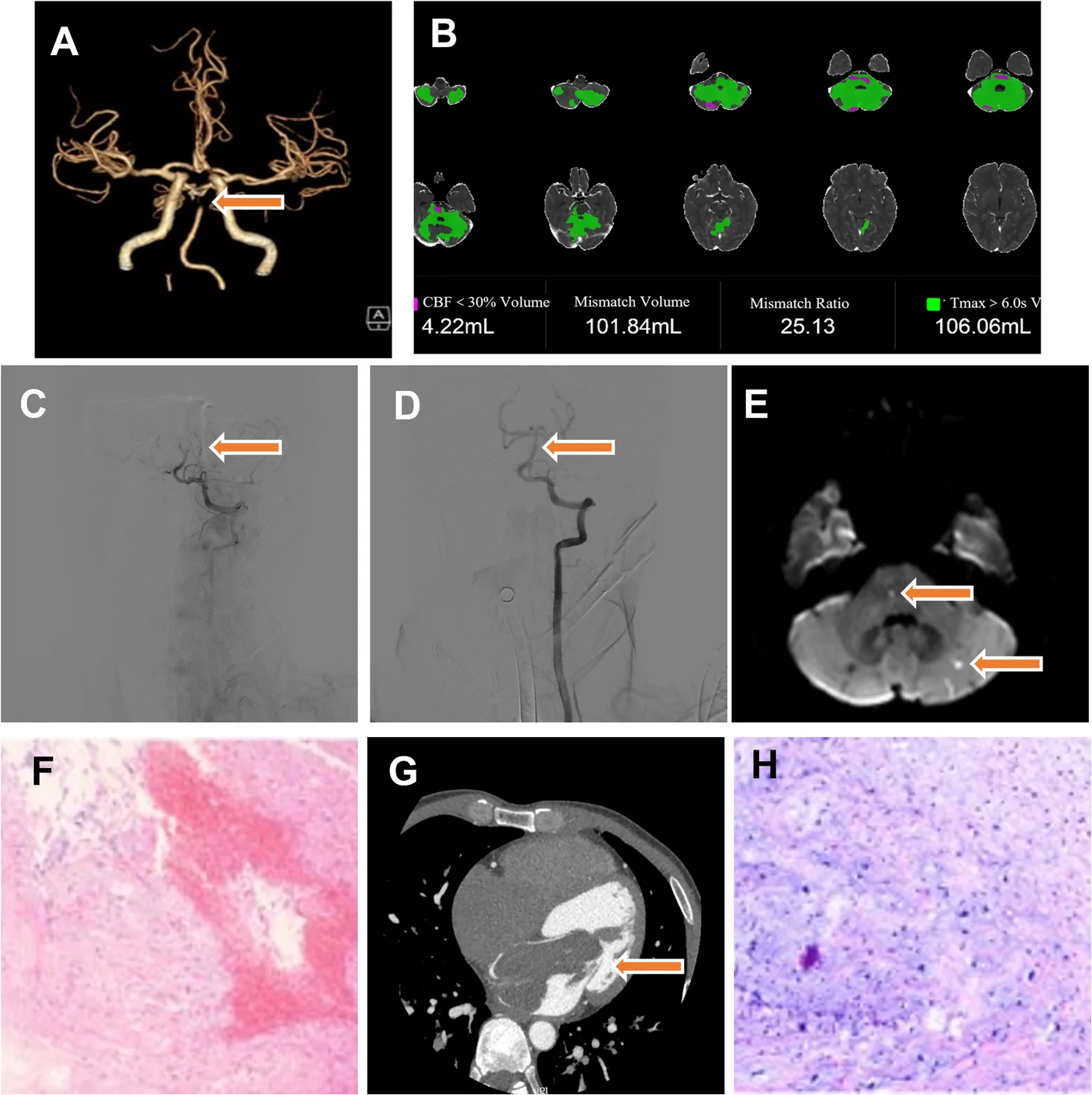
Relevant information for patient 1. Craniocerebral CTA indicating basilar artery occlusion (**A**). Cerebral perfusion imaging suggesting the presence of an ischemic penumbra (**B**). Preoperative Digital Subtraction Angiography (DSA) showing basilar artery occlusion (**C**), postoperative imaging confirming recanalization with an mTICI grade 3 (**D**). Cranial Diffusion-Weighted Imaging (DWI) on postoperative day 1 showing acute cerebral infarction in the brainstem and cerebellum (**E**). Histopathology of the retrieved embolus shows coagulated blood and fibrinous material without atypical cells, confirming thrombotic origin(200×) (**F**). Cardiac CT enhancement suggesting an atrial myxoma (**G**), pathological examination of the resected cardiac tumor confirming atrial myxoma(200×) (**H**)

Mechanical thrombectomy (MT) was initiated 11 h after symptom onset, achieving complete recanalization, defined as a modified Treatment in Cerebral Ischemia (mTICI) score of 3, within 30 min (Fig. 1C–D).

Magnetic resonance imaging (MRI) after the procedure showed acute cerebral infarction in the brainstem and cerebellum without hemorrhage (Fig. 1E). Histopathological examination of the retrieved intracranial embolus revealed thrombotic material admixed with sparse fibrinous material. The absence of atypical cells supports the diagnosis of a thrombotic embolus. (Fig. 1F).

A comprehensive evaluation of stroke etiology identified a large left atrial mass (measuring 73 × 36 mm) on echocardiography, which was confirmed to be a myxoma via cardiac CT and MRI (Fig. 1G). We completed a standardized etiological workup for young-onset ischemic stroke, including transthoracic echocardiography, prolonged ambulatory electrocardiogram monitoring, thrombophilia panel screening, aortic arch and supra-aortic computed tomography angiography (CTA), serum lipid profiling, glycated hemoglobin assay, vasculitis autoantibody screening, and serum homocysteine measurement. No abnormal findings were detected across all tests, leaving atrial myxoma as the sole identifiable stroke etiology. The patient underwent surgical resection. Gross examination revealed a mass with hemorrhagic areas measuring 8 × 6 × 2 cm. Histopathological examination confirmed atrial myxoma with stromal hemorrhage (Fig. 1H).

At the 3-month follow-up, the patient demonstrated excellent functional recovery (Modified Rankin Scale (mRS) score of 1) with preserved cardiac function.

### Patient 2 case presentation

A healthy 19-year-old man experienced sudden-onset dizziness that progressed to loss of consciousness and generalized convulsions whilst playing basketball. After transfer between facilities, the patient received intravenous alteplase (0.9 mg/kg) 3.5 h after the onset of symptoms, which did not result in a significant improvement.

After 7 h of symptom onset, the patient was transferred to our stroke center. Neurological examination revealed coma, with a standard NIHSS score of 33 (I1a = 3, I1b = 2, I1c = 2, I2 = 2, I4 = 3, I5a = 4, I5b = 4, I6a = 4, I6b = 4, I8 = 2, and I9 = 3). Emergency CTA revealed occlusion of the left internal carotid artery and severe stenosis of the right middle cerebral artery, which was confirmed by emergency MT (Fig. 2A, C). Transthoracic echocardiography showed a cardiac mass suggestive of myxoma (Fig. 3A). On physical examination, pedal pulses were absent, indicating an acute peripheral embolism.

**Fig. 2:**
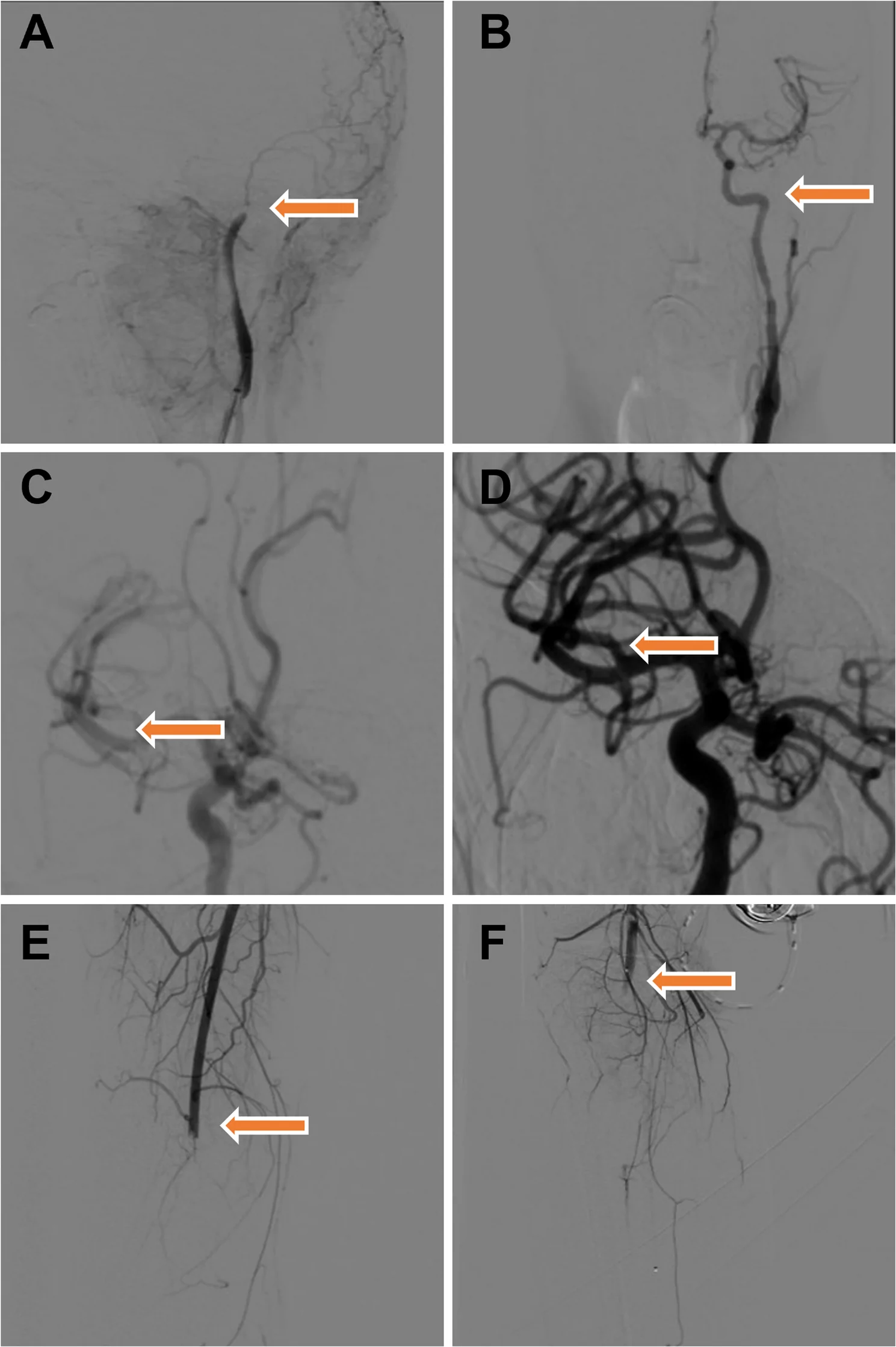
Partial information for patient 2. Intraoperative DSA confirming a left internal carotid artery occlusion and right middle cerebral artery stenosis (**A**, **C**). Lower-limb DSA indicating an embolism in the left femoral artery (**E**). Postoperative DSA showing restored patency of the left internal carotid and right middle cerebral arteries with an mTICI grade 3 (**B**, **D**). Partial removal of the emboli in the lower limb arteries (**F**)

**Fig. 3:**
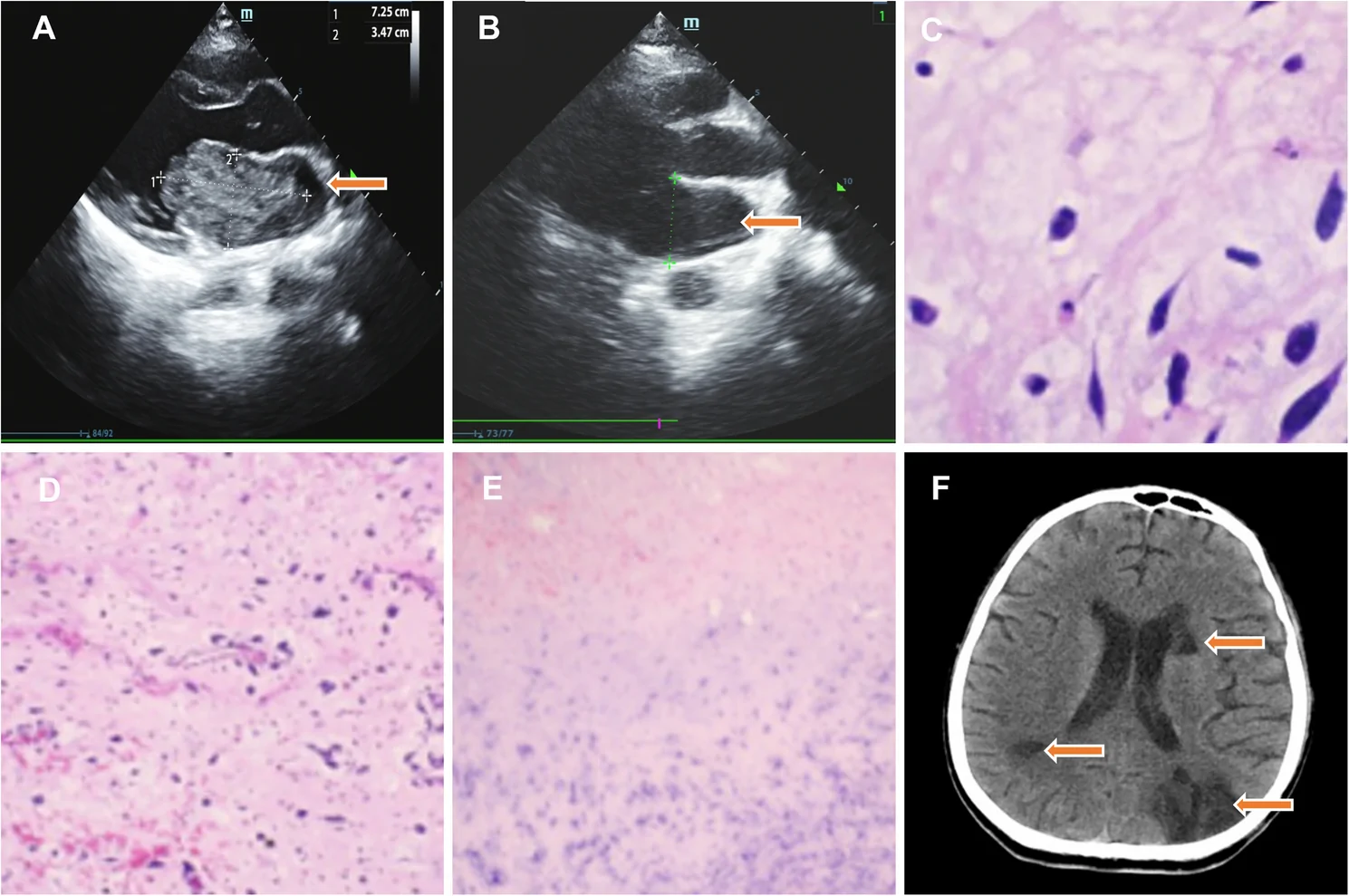
Additional details for patient 2. Echocardiography suggesting a possible atrial myxoma (**A**). Postoperative cardiac ultrasound confirming the absence of tumors (**B**). Specimens harvested from the middle cerebral artery (**C**) and lower limb arteries (**D**) show myxoid stroma and scattered stellate tumour cells with CR immunoreactivity(200×), consistent with atrial myxoma emboli, and atrial myxoma in the resected cardiac tumor (200×) (**E**). Cranial CT demonstrating low-density lesions in both anterior and posterior circulation territories (**F**)

The patient underwent emergent MT 8 h after the symptom onset, resulting in successful vascular recanalization (mTICI grade 3) (Fig. 2B, D), immediately followed by bilateral lower limb thrombectomy. Embolism of the right popliteal and left femoral arteries was confirmed during the procedure (Fig. 2E). After partial embolus retrieval, the family declined further intervention due to technical difficulty (Fig. 2F). Histopathological examination of embolic specimens retrieved from the middle cerebral artery and lower-limb arteries revealed myxoid stroma with scattered stellate-shaped cells. Tumour cells stained positive for calretinin (CR), confirming emboli originating from cardiac atrial myxoma.(Fig. 3C, D).

A standard etiological workup was completed for young stroke, with no alternative etiology identified other than atrial myxoma. The patient received anticoagulation therapy and subsequently underwent successful resection of the cardiac tumor (Fig. 3B), which was confirmed to be an atrial myxoma (Fig. 3E). Follow-up cranial CT at 1 month showed low-density lesions in both anterior and posterior circulation supply areas (Fig. 3F). At 3-month follow-up, the patient achieved substantial functional recovery, characterized by an mRS score of 2.

## Discussion

These cases of stroke provide clear pathological evidence supporting two embolic mechanisms associated with cardiac myxoma, a distinction typically described only in theoretical textbooks. These findings also offer important clinical lessons regarding stroke in young adults and embolic strokes of undetermined origin.

Cardiac myxomas, the most common primary tumor of the heart, possess specific histological characteristics, such as a soft gelatinous matrix containing polymorphic cells and surface fibrin deposition. Combined with the tumor’s mobility and typical left atrial location near the fossa ovalis, these features contribute to mechanical blood-flow stress, which can increase the risk of embolic complications. Embolic events represent the second most common clinical presentation of atrial myxoma, establishing it as a significant etiology of stroke among young adults [3]. Systematic evaluation of potential etiologies in both patients revealed suspected atrial myxoma, and before surgical intervention both cases were classified as cardioembolic according to the TOAST criteria [4]. To reduce recurrence risk [5], both patients underwent tumor resection. MT contributed to cerebral reperfusion [6] and provided embolic tissue for pathological confirmation, a crucial yet often overlooked diagnostic opportunity. Pathological analysis identified distinct mechanisms.

In patient 1, after systematically excluding other common cardioembolic and arterioembolic sources, the thrombotic embolus was suspected to originate from the surface of the atrial myxoma, where endothelial injury and blood flow stasis promoted secondary thrombus formation. This pathophysiology-based anticoagulation therapy allowed for elective surgical timing after neurological stabilization. This approach suggests that while atrial myxoma resection remains the definitive treatment to prevent recurrent embolism, the timing of the procedure can be optimized based on the embolic mechanism. The thrombotic nature allowed delayed intervention, optimizing patient condition before surgical intervention.

In contrast, in patient 2, myxomatous tissue was identified in both cerebral and peripheral emboli, confirming direct tumor fragmentation with minimal thrombotic contribution. This mechanism might contribute to the poor response to intravenous thrombolysis in this patient [7], although large-vessel occlusion itself is associated with decreased efficacy of thrombolysis regardless of embolus composition. Moreover, the coincidence of cerebral and systemic arterial occlusions strongly suggests tumor embolism, serving as a powerful clinical marker that should elevate suspicion for tumor fragmentation rather than pure thromboembolism [8]. Such findings require urgent surgical evaluation and multidisciplinary consultation.

Since the embolus consisted of tumor fragments, follow-up should monitor not only the complications of ischemic stroke but also hemorrhagic transformation after acute infarction, aneurysm formation caused by atrial myxoma, and subarachnoid hemorrhage [9, 10].

Collectively, these cases highlight the need for a multidisciplinary workflow. From the emergency department to neurointerventional, vascular, and cardiac surgery departments, coordinated action is essential to achieving the best outcomes.

## Limitation

This study was limited by its small sample size (only two cases), which undermined the generalizability of our findings. Larger cohort studies are needed to validate the clinical implications of these findings in differentiating between these two embolic mechanisms, particularly in terms of secondary prevention strategies and surgical timing recommendations.

## Conclusion

Atrial myxoma-induced strokes involve two distinct embolic mechanisms that may affect secondary prevention and follow-up strategies. Accurate differentiation relies on the pathological examination of embolic materials, which requires coordinated multidisciplinary care to develop personalized treatment.

## Learning points

- Histopathologic analysis of embolic materials retrieved during MT is imperative to distinguish thrombotic emboli from tumor-fragment emboli in atrial myxoma-related stroke, as the two mechanisms necessitate different clinical management strategies.
- Intravenous thrombolysis is often ineffective for tumor-fragment emboli, underscoring the importance of MT as the primary revascularization strategy for large-vessel occlusion.
- A multidisciplinary approach is the cornerstone of success, integrating stroke neurology, neurointervention, vascular surgery, and cardiac surgery from acute treatment to definitive care.

## Data Availability

The clinical imaging, pathological, and datasets generated and analysed during the current study are not publicly available owing to patient privacy restrictions. All key imaging and pathological findings supporting our conclusions are presented in the figures and main text of this manuscript. Anonymized raw data can be obtained from the corresponding author upon reasonable request, subject to institutional ethics approval.

## Abbreviations

CT: Computed tomography
CTA: Computed tomography angiogram
RAPID: RApid processing of PerfusIon and Diffusion
MT: Mechanical thrombectomy
MRI: Magnetic resonance imaging
NIHSS: National Institutes of Health Stroke Scale
mTICI: Modified treatment in cerebral ischemia
DSA: Digital subtraction angiography
DWI: Diffusion-weighted imaging
CR: Calretinin
